# Supplementary material for: Safety and Efficacy of Sodium and Potassium Arachidonic Acid Salts in the Young Pig
Source: Nutrients. 2021 Apr 27;13(5):1482. doi: 10.3390/nu13051482 (PMC8145490; doi:10.3390/nu13051482)
Supplement: Supplementary file 1 [file nutrients-13-01482-s001.zip › nutrients-1193501-supplementary.pdf]

## SUPPLEMENTARY MATERIALS

### 1. *Diet analyses*

When provided in the TG form, both ARA and DHA were analyzed to contain the correct formulated concentrations, suggesting there were no issues with diet manufacturing. All diet samples were collected shortly after initiating the live-animal study and stored in airtight glass containers away from light and at -20°C until analysis. Given these quality controls to preserve the integrity of the diet samples, we have no definitive evidence to suggest the test articles degraded.

**Table S1.** ARA and DHA content of MR powders (mg/g)[1]

| | Treatment | TT | NaT | KT | Na0 | NaNa |
|---|---|---|---|---|---|---|
| | **ARA** | **TG** | **Na-salt** | **K-salt** | **Na-salt** | **Na-salt** |
| **Item** | **DHA** | **TG** | **TG** | **TG** | **-** | **Na-salt** |
| ARA | | 0.82 | 1.15 | 0.21 | 0.19 | 0.02 |
| DHA | | 0.51 | 0.49 | 0.52 | ND | ND |

[1]Diets were manufactured as custom blends formulated by TestDiet (St. Louis, MO). All pigs received allotted treatment from PND 2 to PND 23. Abbreviations: ARA, arachidonic acid; DHA, docosahexaenoic acid; MR, milk replacer; ND, not detectable; TG, triglyceride. Values are numerical averages from three independent analyses. Samples were analyzed in duplicate or greater within each run for a total of eight individual sample analyses.

### 2. *Liver Histology*

**Table S2.** Liver histology of pigs receiving experimental milk replacers with different sources of ARA[1]

| | Diet | TT | NaT | KT | Na0 | NaNa | Pooled | Model |
|---|---|---|---|---|---|---|---|---|
| **Outcome** | **ARA/DHA[2]** | **0.47/0.32** | **0.47/0.32** | **0.47/0.32** | **0.47/0.00** | **0.47/0.32** | **SEM** | ***P*-value** |
| Necrosis | | ND | ND | ND | ND | ND | - | - |
| Lipid[3] | | 0.50 | 0.09 | 0.05 | 0.25 | 0.21 | 0.259 | 0.386 |
| Vacuolization[4] | | 3.50 | 3.50 | 3.42 | 3.31 | 2.91 | 0.278 | 0.526 |
| Inflammation | | 0.42 | 0.25 | 0.17 | 0.30 | 0.10 | 0.139 | 0.442 |
| Biliary | | 0.50 | 0.42 | 0.49 | 0.52 | 0.09 | 0.167 | 0.169 |
| Fibrosis | | ND | ND | ND | ND | ND | - | - |
| EMH | | 1.34 | 1.34 | 1.82 | 1.52 | 1.47 | 0.243 | 0.383 |

[1]Values represent least square means of 11-13 pigs per treatment group at PND 23. Abbreviations: ARA, arachidonic acid; DHA, docosahexaenoic acid; EMH, extra medullary hematopoiesis; ND, not detected; SEM, standard error of the mean.
[2]Formulated dietary ARA and DHA content (% of total fatty acids).
[3]Scored using the following scale: 1 = mild, 2 = moderate, 3 = marked, 4 = severe.
[4]Score using the following scale: 1 = minimal, 2 = mild, 3 = moderate, 4 = marked.
